# Spontaneous Splenic Vein Rupture: Case Report of a Rare Presentation

**DOI:** 10.5811/cpcem.47345

**Published:** 2026-01-13

**Authors:** Brittany Franco, Amanda Karski, Kevin Boehm

**Affiliations:** Broward Health North Hospital, Department of Emergency Medicine, Deerfield Beach, Florida

**Keywords:** splenic vein, spontaneous rupture, retroperitoneal hemorrhage, case report

## Abstract

**Introduction:**

Spontaneous splenic vein rupture is a rare condition associated with a high risk of mortality. Only a few cases have been documented, all of which have been linked to underlying predisposing conditions. In this case, however, we present a previously healthy patient with no identifiable risk factors.

**Case Report:**

A 64-year-old male presented to the emergency department with acute-onset abdominal pain and nausea. Physical exam revealed a rigid abdomen with diffuse tenderness and guarding. Serial labs revealed a progressively decreasing hemoglobin and red blood cell count. Computed tomography demonstrated a ruptured splenic vein with a large volume of retroperitoneal hemorrhage. Laparotomy identified a large retroperitoneal hematoma with hemoperitoneum, and embolization was performed by interventional radiology. The patient recovered after one week in the surgical intensive care unit and was subsequently discharged home with no complications to date.

**Conclusion:**

This case underscores the importance of maintaining a high clinical suspicion in patients with acute, unexplained abdominal pain, and emphasizes the crucial role of emergency physicians in the timely recognition and management of such conditions. Given the scarcity of existing literature, this case provides valuable insight into the presentation and management of spontaneous splenic vein rupture in previously healthy individuals, thereby enhancing clinical awareness of this rare and life-threatening condition.

## INTRODUCTION

Spontaneous splenic vein rupture is a rare and potentially life-threatening condition characterized by the sudden rupture of the splenic vein, leading to significant retroperitoneal hemorrhage and hypovolemic shock in the absence of preceding trauma. The etiology remains unclear; however, predisposing conditions may include splenic vein aneurysm or thrombosis, liver cirrhosis, and pregnancy.^1^–^5^ In this report we present a rare case of spontaneous splenic vein rupture in a previously healthy patient with no identifiable predisposing factors. With only a few documented cases in the medical literature, recognizing this condition requires a high index of suspicion when evaluating patients with unexplained abdominal pain. This case underscores the critical role of emergency physicians in the timely identification and management of spontaneous vascular injuries to prevent potentially fatal complications.

## CASE REPORT

A 64-year-old male with a medical history of hypertension, hyperlipidemia, and benign prostatic hyperplasia presented to the emergency department (ED) for acute-onset abdominal pain and nausea for three hours. The patient stated that he had been bicycling earlier that day and explicitly denied any trauma, including a bicycle accident or handlebar injury. Social history was negative for tobacco, alcohol, or illicit drug use. He denied fever, chills, vomiting, diarrhea, constipation, melena, hematochezia, hematemesis, dysuria, hematuria, syncope, or weakness. Upon arrival to the ED, his vital signs were as follows: heart rate, 76 beats per minute; blood pressure, 124/85 millimeters of mercury; oxygen saturation, 94% on room air; and temperature, 97.9 ° Fahrenheit. Neurologic examination was non-focal. The mucosa was pink and moist, and there was no conjunctival pallor. Cardiopulmonary examination was unremarkable. Pulses were equal in bilateral upper and lower extremities. Abdominal exam revealed a rigid abdomen with diffuse tenderness, more pronounced in the epigastrium and left upper quadrant, and with involuntary guarding. There was no costovertebral angle, tenderness, and McBurney and Murphy signs were negative.

Initial labs included a complete blood count (CBC), comprehensive metabolic panel, lipase, troponin, magnesium, type and screen, prothrombin time and international normalized ratio (INR), Thromboelastogram, and urinalysis. CBC was remarkable for mild normocytic anemia (hemoglobin 12.8 grams per deciliter (g/dL) (reference range: 13.0–17.3 g/dL) without leukocytosis or thrombocytopenia. Repeat CBC (three hours later), was notable for a decrease in hemoglobin to 10.9 g/dL. Comprehensive metabolic panel, lipase, and troponin were unremarkable. The INR was 1.3. thromboelastogram, demonstrated a shortened R-time of 3.7 minutes (4.6–9.1 minutes) but was otherwise normal.

**Image 1:**
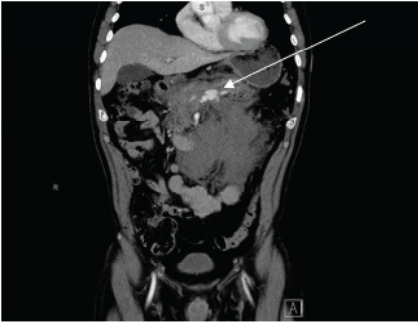
Computed tomography abdomen and pelvis demonstrating abnormal dilatation of the splenic vein (arrow) with large volume surrounding fluid of varying densities, suggesting splenic vein rupture with extensive retroperitoneal hemorrhage.

**Image 2:**
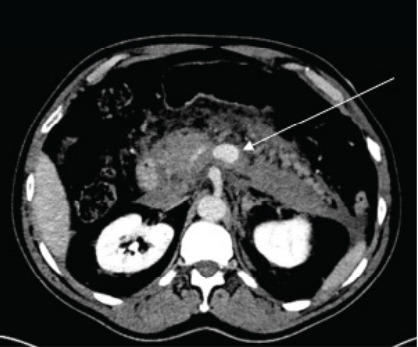
Computed tomography angiogram abdomen and pelvis during the delayed venous phase demonstrating a large amount of hyperdense material surrounding the pancreas extending into the left anterior perirenal space. A focal contrast blush is visible just anterior to the celiac artery (arrow).

Initial computed tomography (CT) of the abdomen and pelvis with intravenous contrast demonstrated abnormal dilatation of the splenic vein with large volume surrounding fluid of varying densities, suggesting splenic vein rupture with extensive retroperitoneal hemorrhage (Image 1). Computed tomography of the abdomen and pelvis with venous and delayed venous phases was subsequently ordered and confirmed splenic vein rupture at the level of the mid-pancreatic body with active extravasation (Image 2). There was no evidence of splenic artery aneurysm or other vascular anomaly noted (Video).

*CPC-EM Capsule*

What do we already know about this clinical entity?

*Spontaneous splenic vein rupture is rare, usually linked to trauma, cirrhosis, thrombosis, or pregnancy.*

What makes this presentation of disease reportable?

*A previously healthy patient with no identifiable risk factors presented with spontaneous splenic vein rupture.*

What is the major learning point?

*Maintain high suspicion for spontaneous vascular rupture in patients with acute abdominal pain, even when they are stable and without risk factors.*

How might this improve emergency medicine practice?

*Awareness of spontaneous vascular rupture in unexplained abdominal pain will enable clinicians to play a critical role in early imaging and timely intervention.*

The patient initially received one liter of lactated Ringer’s, ondansetron, famotidine, and morphine without resolution of his symptoms. He grew increasingly uncomfortable, prompting the administration of hydromorphone. After receiving the results of the CT abdomen and pelvis, urgent surgical and interventional radiology consultations were placed. The patient was admitted to the surgical intensive care unit (SICU) in stable condition. Given the venous and retroperitoneal nature of the bleed and the patient’s stable hemodynamic status at the time of admission, the surgical team opted for conservative management and close monitoring to allow tamponade in the retroperitoneum. However, the patient’s hemoglobin levels continued to decline, and his clinical status deteriorated, necessitating emergent surgical intervention.

Emergent laparotomy revealed a large retroperitoneal hematoma with hemoperitoneum. The patient was stabilized after receiving two units of packed red blood cells and fresh frozen plasma. Interventional radiology then performed an embolization of an un-named branch of the right gastroepiploic artery. The abdomen was irrigated and left open with a wound vacuum-assisted closure in place. The patient was transferred back to the SICU in stable condition.

Two days later, he was returned to the operating room for a reopening laparotomy, washout, and wound closure. This operation revealed a stable, non-pulsatile retroperitoneal hematoma without active extravasation. No additional abdominal exploration was performed. The abdomen was then irrigated, and the wound was closed. The patient tolerated this procedure well, requiring no additional blood product administration.

He remained hemodynamically stable throughout his SICU stay. After 10 days of steady recovery with stable hemoglobin levels and no complications, he was discharged home with a structured outpatient management plan and surgical follow-up. To date, the patient has had no reported complications.

## DISCUSSION

Splenic vein rupture with subsequent intra-abdominal hemorrhage is a rare condition, typically associated with trauma.^6^ This case presents a patient with spontaneous splenic vein rupture occurring without trauma or underlying predisposing factors, providing valuable insight into this unique condition. The exact pathophysiology of spontaneous splenic vein rupture remains uncertain, although reported risk factors include splenic vein aneurysm or thrombosis, liver cirrhosis, and pregnancy.^1^–^5^ Documented examples include rupture of a splenic vein aneurysm during pregnancy^1^ and splenic vein rupture in the setting of liver cirrhosis,^2^ both resulting in massive intra-abdominal hemorrhage. In such cases, the proposed mechanism is largely attributed to portal hypertension. In pregnancy, the combination of mechanical compression of the inferior vena cava and portal system by the gravid uterus, increased splanchnic venous pressure due to pregnancy-induced hemodynamic changes, and hormonal alterations—particularly, elevated estrogen and progesterone— remodeling can compromise the vessel wall integrity and increase susceptibility to rupture.^3^–^4^

In patients without predisposing risk factors, as demonstrated in this case report, the underlying cause of rupture remains unknown. The rarity of spontaneous splenic vein rupture in the absence of underlying pathology cannot be emphasized enough. Of the documented cases, the clinical presentation includes acute abdominal pain, hypotension, and signs of hemorrhagic shock.^1^–^5^ This may reduce the suspicion of intra-abdominal hemorrhage in hemodynamically stable patients as seen in this case. This highlights the necessity to maintain a high index of suspicion for spontaneous vascular rupture in seemingly healthy patients with acute-onset abdominal pain in the absence of hemodynamic instability.

Imaging studies are crucial for diagnosis. Ultrasound and CT are the primary modalities used to identify intra-abdominal hemorrhage and vascular pathology.^7^,^9^ Point-of-care ultrasound (POCUS) enables rapid identification of intraperitoneal free fluid.^7^ Despite its clinical utility, POCUS has limitations, including reduced sensitivity in detecting retroperitoneal injuries and its reliance on the volume of free fluid for accurate detection. Experienced operators may detect volumes as low as 200 milliliters (mL), but the average volume required for detection is 619 mL.^8^ Computed tomography, specifically angiogram with venous and delayed venous phases, can provide detailed images to confirm the presence of a splenic vein rupture, identify the source of bleeding, and assess the extent of hemoperitoneum.^9^

The management of spontaneous splenic vein rupture is largely dependent on the patient’s hemodynamic status. Conservative treatment is appropriate for hemodynamically stable patients, but surgical intervention is necessary in cases of instability or rapid decompensation.^10^ In this case, the patient was initially managed conservatively, but as his condition deteriorated, laparotomy and embolization via interventional radiology were necessary for stabilization.

## CONCLUSION

Spontaneous splenic vein rupture is a rare but potentially fatal condition characterized by the sudden rupture of the splenic vein, leading to significant intra-abdominal hemorrhage and shock. Few cases of splenic vein rupture have been reported, all of which have been linked to underlying predisposing conditions. This case report contributes to the limited body of research on spontaneous splenic vein rupture in previously healthy individuals with no identifiable predisposing factors. It emphasizes the importance of maintaining a high clinical suspicion when evaluating patients with acute, unexplained abdominal pain. This case highlights the critical role of emergency physicians in the prompt recognition and management of spontaneous vascular injuries to improve patient outcomes and prevent further morbidity and mortality.

## Figures and Tables

**Video:** Computed tomography angiogram of the abdomen and pelvis demonstrating no evidence of splenic artery aneurysms (arrow) or other vascular anomaly.
